# The P300 Auditory Event-Related Potential May Predict Segregation of Competing Speech by Bimodal Cochlear Implant Listeners

**DOI:** 10.3389/fnins.2022.888596

**Published:** 2022-06-10

**Authors:** Duo-Duo Tao, Yun-Mei Zhang, Hui Liu, Wen Zhang, Min Xu, John J. Galvin, Dan Zhang, Ji-Sheng Liu

**Affiliations:** ^1^Department of Ear, Nose, and Throat, Shaanxi Provincial People's Hospital, Xi'An, China; ^2^Department of Ear, Nose, and Throat, The First Affiliated Hospital of Soochow University, Suzhou, China; ^3^House Institute Foundation, Los Angeles, CA, United States

**Keywords:** cochlear implant, competing speech, informational masking, event-related potentials, P300

## Abstract

Compared to normal-hearing (NH) listeners, cochlear implant (CI) listeners have greater difficulty segregating competing speech. Neurophysiological studies have largely investigated the neural foundations for CI listeners' speech recognition in quiet, mainly using the P300 component of event-related potentials (ERPs). P300 is closely related to cognitive processes involving auditory discrimination, selective attention, and working memory. Different from speech perception in quiet, little is known about the neurophysiological foundations for segregation of competing speech by CI listeners. In this study, ERPs were measured for a 1 vs. 2 kHz contrast in 11 Mandarin-speaking bimodal CI listeners and 11 NH listeners. Speech reception thresholds (SRTs) for a male target talker were measured in steady noise or with a male or female masker. Results showed that P300 amplitudes were significantly larger and latencies were significantly shorter for the NH than for the CI group. Similarly, SRTs were significantly better for the NH than for the CI group. Across all participants, P300 amplitude was significantly correlated with SRTs in steady noise (*r* = −0.65, *p* = 0.001) and with the competing male (*r* = −0.62, *p* = 0.002) and female maskers (*r* = −0.60, *p* = 0.003). Within the CI group, there was a significant correlation between P300 amplitude and SRTs with the male masker (*r* = −0.78, *p* = 0.005), which produced the most informational masking. The results suggest that P300 amplitude may be a clinically useful neural correlate of central auditory processing capabilities (e.g., susceptibility to informational masking) in bimodal CI patients.

## Introduction

While cochlear implants (CIs) provide sufficient spectro-temporal resolution for speech recognition in quiet by deaf individuals, masked speech recognition is often difficult for CI users. Steady noise is thought to largely produce “energetic” masking; the spectro-temporal overlap between the target and masker occurs at the periphery (e.g., Brungart, 2001; Kidd et al., 2002). Competing speech is thought to produce some combination of energetic masking, “envelope” masking (target and masker envelope interference even when there is no spectral overlap; e.g., Stone and Canavan, 2016), and “informational” masking (e.g., lexical interference, target/masker similarities, etc.; Brungart, 2001; Kidd et al., 2002, 2016). Different from normal-hearing (NH) listeners, who have greater difficulty with competing noise than with competing speech, CI listeners have greater difficulty with competing speech than with competing noise (e.g., Stickney et al., 2004; Cullington and Zeng, 2008; Tao et al., 2018). The coarse spectro-temporal resolution is thought to limit CI users' segregation of target and masker speech (e.g., Friesen et al., 2001; Shannon et al., 2004; Fu and Nogaki, 2005; Luo and Fu, 2009).

Cortical measures have been used to characterize NH and CI listeners' auditory processing. Auditory event-related potentials (ERPs) reflect the brain's response to changes in an ongoing stimulus (e.g., deviant stimuli in the context of frequent stimuli in an oddball paradigm). Exogenous, pre-attentive responses (e.g., P1, N1, P2, N2 peaks) typically occur within the first 250 ms and do not reflect cognitive processing (e.g., Martin et al., 2008; Lightfoot, 2016). The latency of the endogenous P3 (or P300) response is typically between 250 and 400 ms, and is thought to reflect attention and/or arousal (e.g., Polich and Kok, 1995; Kok, 2001). Recording of P300 responses requires some sort of behavioral response to the deviant stimulus (e.g., counting the number of deviant stimuli during a test run, indicating when a deviant stimulus was heard, etc.). P300 latency has been shown to be related to the speed of information processing (e.g., Ritter et al., 1972; Kutas et al., 1977; Parasuraman and Beatty, 1980; Donchin and Coles, 1988). P300 amplitude has been shown to decrease with increasing task difficulty (e.g., Parasuraman and Beatty, 1980). Uncertainty in discrimination of sounds may be reflected in reduced P300 amplitude (e.g., Sutton et al., 1965; Hillyard et al., 1971; Squires et al., 1973; Picton, 2011).

There is great variability in CI outcomes that is largely unexplained but may be related to individual central auditory processing capacities (e.g., Dunn et al., 2005). In CI users, ERPs may be used to observe detection (exogenous components) and discrimination (endogenous components) of stimulus contrasts. Assuming there are no cognitive deficits, device-related factors (e.g., the number of implanted electrodes, frequency allocation) and patient-related factors (e.g., the electrode-neural interface, patterns of neural survival, etc.) may affect ERP responses. As such, it is important to select stimuli that are sufficiently contrastive when measuring ERPs. Some studies have used pure-tone contrasts (e.g., Groenen et al., 2001; Beynon et al., 2002; Sasaki et al., 2009; Obuchi et al., 2012; Calderaro et al., 2020; Van Yper et al., 2020; Wedekind et al., 2021) while others have used phonemic contrasts (e.g., Groenen et al., 2001; Beynon et al., 2002, 2005; Beynon and Snik, 2004; Henkin et al., 2009; Micco et al., 1995). ERPs have also been used to observe the evolution of auditory processing after cochlear implantation in longitudinal studies (e.g., Kubo et al., 2001).

P300 is closely related to cognitive processes involving auditory discrimination, selective attention, and working memory (e.g., Polich, 2007). Segregation of competing speech has been shown to involve cognitive processes (e.g., Francis, 2010). Some CI studies have compared P300 responses to standard clinical measures such as word recognition in quiet (e.g., Kileny et al., 1997; Groenen et al., 2001; Grasel et al., 2018; Abrahamse et al., 2021; Amaral et al., 2021). Others have compared P300 responses to phoneme recognition in quiet (e.g., Groenen et al., 2001; Beynon et al., 2002) or to speech recognition in steady noise (e.g., Iwaki et al., 2004). Kileny et al. (1997) found a significant correlation between P300 amplitude and sentence recognition in pediatric CI users. Groenen et al. (2001) found a significant correlation between P300 amplitude and word/phoneme recognition in quiet in adult CI users.

Bimodal listening [CI in one ear, hearing aid (HA) in the other ear] provides important low-frequency temporal fine-structure cues that benefit pitch-mediated perception (e.g., music, talker identity, prosody) and segregation of target speech and maskers (e.g., Gifford et al., 2007; Cullington and Zeng, 2008; Dorman et al., 2008; Yoon et al., 2012; Crew et al., 2015; Liu et al., 2019). Previous studies have shown more robust P300 responses with bimodal than with CI-only listening. Iwaki et al. (2004) found that sentence recognition in noise was significantly better and P300 latency was significantly shorter with bimodal than with CI-only listening. Sasaki et al. (2009) also reported shorter P300 latency and better word recognition in quiet with bimodal than with CI-only listening. However, the relationship between P300 responses and segregation of competing speech with bimodal listening remains unclear.

In this study, P300 responses to pure-tone stimuli were recorded in NH listeners and bimodal CI users; speech recognition was measured in the presence of steady noise or competing speech. Given that the present participants used bimodal listening in daily life, only bimodal listening was tested. Also, previous studies have shown more robust P300 responses with bimodal than with CI-only listening (e.g., Iwaki et al., 2004; Sasaki et al., 2009). Consistent with previous studies (e.g., Beynon et al., 2005; Obuchi et al., 2012; Grasel et al., 2018), we expected greater P300 amplitudes and shorter P300 latencies in NH than in CI listeners. Given the great variability in speech performance among CI users (e.g., Stickney et al., 2004; Cullington and Zeng, 2008) and given that P300 is sensitive to auditory task difficulty (Parasuraman and Beatty, 1980; Polich, 1987; Causse et al., 2016), we expected that P300 responses would be related to masked speech recognition, especially for the more difficult segregation of competing speech by CI users.

## Methods

### Participants

Eleven Mandarin-speaking CI listeners (six females, five males) participated in the study; the mean age at testing was 21.5 ± 9.2 years. All were users of Med-El devices. All except for CI-4 were implanted with the Sonata ti10 device with the Standard electrode array (31.5 mm); CI-4 was implanted with Concerto device and the Flex 28 electrode array (28 mm). All used the Opus 2 processor, and all used the FS4 strategy. All were bimodal listeners, using a CI in one ear and a hearing aid in the other ear in every day listening. The mean duration of deafness prior to implantation was 12.8 ± 6.5 years. The mean CI experience was 2.0 ± 1.9 years. CI participants C1, C2, A2, A3, A6 were prelingually deaf, and C3, A1, A4, A5, A7, A8 were postlingually deaf. Table 1 shows demographic information for the CI participants. Eleven Mandarin-speaking NH listeners (seven females, four males) also participated in the study; the mean age at testing was 22.1 ± 9.5 years. A *t*-test showed no significant difference in age at testing between the CI and NH groups [*t*(20) = 0.2 *p* = 0.882]. All participants were recruited from Department of Ear, Nose, and Throat, The First Affiliated Hospital of Soochow University. The Ethical Committee from The First Affiliated Hospital of Soochow University specifically approved this study (Approval number 2021122). All participants provided written informed consent before participating in the study; parental approval was obtained for pediatric CI and NH listeners.

**Table 1:** Demographic information of CI participants.

**Participant**	**Sex**	**Age at test** **(yrs)**	**Dur deaf** **(yrs)**	**CI exp** **(yrs)**	**CI ear**	**Etiology**	**PTA** **(dB HL)**	**Mean HA gain** **(dB)**
CI-C1	M	6.5	3.4	3.3	R	Congenital	94.2	33.3
CI-C2	F	10.6	8.0	0.8	L	Congenital	81.7	40.8
CI-C3	F	14.9	7.9	0.5	R	Unknown	82.5	29.2
CI-A1	F	20.1	15.0	0.8	R	Progressive	91.7	38.3
CI-A2	F	20.3	20.3	3.5	R	Congenital	90.8	29.2
CI-A3	F	20.7	20.0	0.6	L	Congenital	85.8	25.0
CI-A4	M	23.8	14.8	1.2	L	Unknown	85.0	23.3
CI-A5	M	23.9	12.9	1.0	L	Unknown	69.2	22.5
CI-A6	M	24.1	21.0	6.7	R	Congenital	81.7	20.0
CI-A7	M	31.3	15.0	0.6	R	Progressive	86.7	41.7
CI-A8	F	40.2	2.5	2.5	R	Sudden	83.3	17.5

*All participants were users of Med-El devices, and all were everyday bimodal listeners (CI in one ear, hearing aid in the other ear)*.

*Unaided PTA thresholds were calculated across 0.25, 0.5, 1.0, 2.0, 4.0, and 8.0 kHz. The mean HA gain was calculated as the mean difference between unaided and aided PTA thresholds. Dur deaf, duration of deafness before cochlear implantation; CI exp, experience with the CI device; CI-C, CI children; CI-A, CI adult*.

### Speech Perception

The Closed-set Mandarin Speech (CMS; Tao et al., 2017) test materials were used to test speech recognition with the different maskers. The CMS test materials consist of familiar words selected to represent the natural distribution of vowels, consonants, and lexical tones found in Mandarin Chinese. Ten keywords in each of five categories (Name, Verb, Number, Color, and Fruit) were produced by native Mandarin talkers.

Speech reception thresholds (SRTs), defined as the target-to-masker ratio (TMR) that produced 50% correct keyword recognition, were adaptively measured using a modified coordinate response matrix test (Brungart, 2001). Two target keywords (randomly selected from the Number and Color categories) were embedded in a five-word carrier sentence uttered by a male target talker [mean fundamental frequency (F0) across all words = 136 Hz]. The first word in the target sentence was always the Name “Xiaowang,” followed by randomly selected words from the remaining categories. Thus, the target sentence could be (translated from Mandarin) “**Xiaowang** sold ***Three Red*** strawberries” or “**Xiaowang** chose ***Four Brown*** bananas,” etc. (Name to cue target talker in bold; target keywords in bold italic).

Recognition of the target keywords was measured in the presence of steady state noise (SSN) or competing speech; maskers were co-located with the target (0° azimuth). The spectrum of the SSN was matched to the long-term average spectrum of the target talker, averaged across all words. For competing speech, the masker was a female talker (mean F0 across all words = 248 Hz) or a different male talker (mean F0 = 178 Hz). Masker sentences were randomly generated for each test trial; words were randomly selected from each category, excluding the words used in the target sentence. Thus, the masker sentence could be “Xiaozhang saw *Two Blue* kumquats,” “Xiaodeng took *Eight Green* papayas,” etc. (competing keywords in italic).

All stimuli were presented in the sound field at 65 dBA *via* a single loudspeaker; subjects were seated in a sound-attenuated booth, directly facing the loudspeaker at a 1-m distance. For CI participants, SRTs were measured using the clinical settings for their devices, which were not changed throughout the study. During each test trial, a sentence was presented at the desired TMR; the initial TMR was 10 dB. Participants were instructed to listen to the target sentence (produced by the male target talker and beginning with the name “Xiaowang”) and then click on one of the 10 response choices for each of the Number and Color categories; no selections could be made from the remaining categories, which were grayed out. If the subject correctly identified both keywords, the TMR was reduced by 4 dB (initial step size); if the subject did not correctly identify both keywords, the TMR was increased by 4 dB. After two reversals, the step size was reduced to 2 dB. The SRT was calculated by averaging the last six reversals in TMR. If there were fewer than six reversals within 20 trials, the test run was discarded and another run was measured. Two test runs were completed for each condition and the SRT was averaged across runs. The masker conditions were randomized within and across participants.

### P300 Recordings

P300 ERPs were recorded using the Smart EP software (Intelligent Hearing System, Miami, FL, USA) and a multichannel recording paradigm. Disposable electrodes were placed at the high forehead (non-inverting electrode), both sides of the mastoid (inverting electrode), and low forehead (ground electrode). Absolute impedances and inter-electrode impedances were <5 and 3 kΩ, respectively. Responses were filtered online using a band-pass filter between 1 and 100 Hz. Pure-tone acoustic stimuli (1 or 2 kHz) with 50-ms duration and 5-ms rise and decay times were presented to the subjects every 1 s. Pure-tone stimuli were used instead of speech stimuli because pure-tone stimuli show better P300 reproducibility (e.g., Perez et al., 2017). The intensity of the stimuli was 20–30 dB above the aided PTA thresholds at 1 or 2 kHz to ensure that stimuli were clearly and comfortably audible for all participants.

Participants were seated in an electrically-shielded, sound-attenuated examination room. The stimuli were presented *via* two loudspeakers placed at ear level, 1 m away, ±45° relative to center. The probability was set at 80% for the frequent stimulus (1 kHz tone) and 20% for the rare stimulus (2 kHz tone). Participants were instructed to count the number of 2 kHz stimuli (oddball paradigm). All participants were able to discriminate between 1 and 2 kHz with 100% accuracy. In each run where all 20 oddball stimuli were identified, 20 ERPs for the rare stimuli were averaged. The recording window was comprised of a pre-stimulus baseline of 200 ms and a 500 ms post-stimulus epoch with a sampling rate of 1,000 Hz. Artifact rejection level was set at 100 mV. To avoid artifacts due to eye blinks, participants were instructed to close their eyes during the recording (Groenen et al., 2001). To reduce unwanted alpha rhythm, the inter-stimulus-interval was jittered by ±0.1 s (±10%), which made stimulus presentation less predictable and participants more attentive. Also, alpha rhythm was partially canceled out during the average processing because the onset of the P300 ERP is random relative to the phase of the alpha wave (Talsma and Woldorff, 2005).

P300 amplitude was calculated between the most positive point in the waveform between ≈250–400 ms and the following most negative point. This approach was chosen because the following most negative point was more distinct than the previous negative point. P300 latency was identified according to the P300 positive point. A minimum of three runs were tested, with more as needed if the participant did not identify all 20 oddball stimuli; only test runs where all 20 oddball stimuli were identified were included in analyses. Rest periods were taken between sessions to keep the participants alert.

## Results

**Figure 1:**
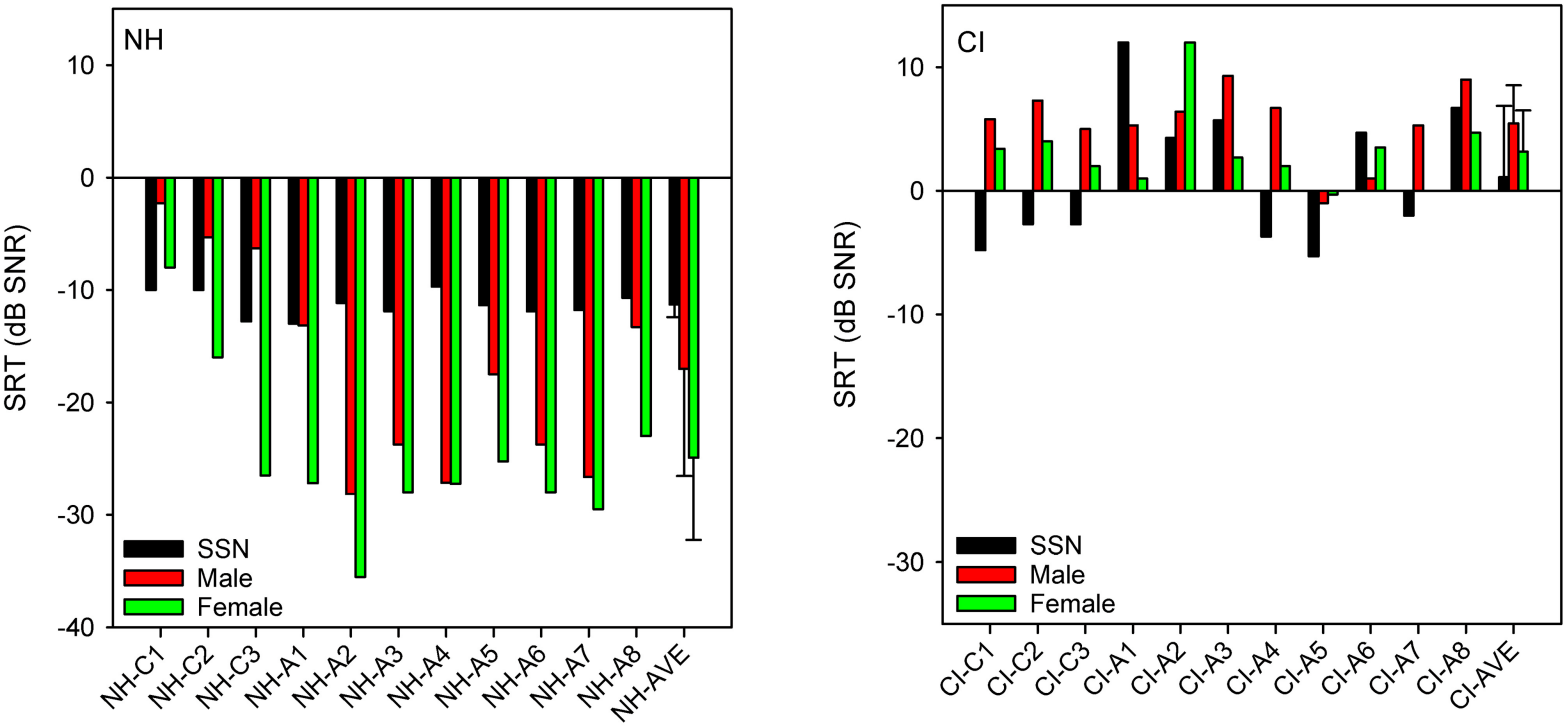
**(Left)**: SRTs with SSN, competing female, or competing masker for individual NH participants; mean SRTs across NH participants are shown at right. **(Right)**: Same as left panel, but for CI participants. In both panels, participants are ordered in terms of age at testing, with “C” indicating child listeners and “A” indicating adult listeners. The error bars show the standard deviation.

Figure 1 shows SRTs with SSN or with a competing male or female talker for the NH and CI listeners. SRTs were much lower (better) for NH than for CI listeners. For NH listeners, mean SRTs progressively improved from SSN (−11.3 ± 1.1 dB) to the male masker (−17.0 ± 9.0 dB) and then to the female masker (−24.9 ± 7.3 dB). For CI listeners, mean SRTs were poorer with the competing male (5.5 ± 3.1 dB) or female talker (3.2 ± 3.3 dB) than with SSN (1.1 ± 5.9 dB). A mixed-design analysis of variance (ANOVA) was performed on the SRT data, with masker (SSN, male, female) as the within-subject factor and group (NH, CI) as the between-subject factor. Results showed significant effects of group [*F*_(1,40)_ = 125.1, *p* < 0.001] and masker [*F*_(2,40)_ = 10.7, *p* < 0.001]; there was a significant interaction [*F*_(2,40)_ = 16.8, *p* < 0.001]. *Post-hoc* Bonferroni pairwise comparisons showed that for the NH group, SRTs were significantly higher (poorer) with SSN than with the male (*p* = 0.016) or female masker (*p* < 0.001), and significantly higher with the male than with the female masker (*p* < 0.001). There were no significant differences among the maskers for the CI group. SRTs were significantly lower (better) for the NH than for the CI group for all maskers (*p* < 0.001 for all comparisons).

**Figure 2:**
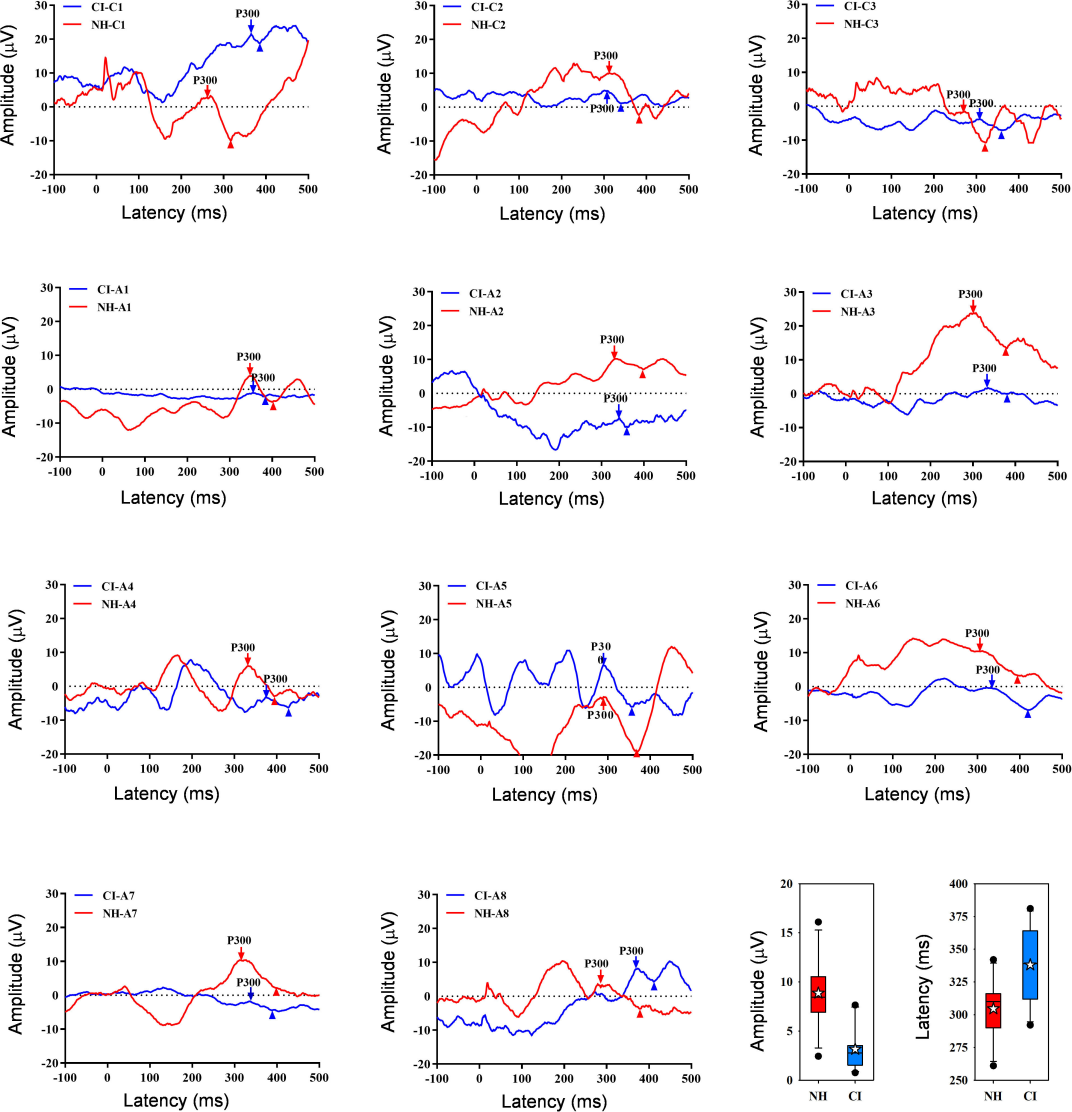
Individual age-matched NH (red) and CI listener (blue) waveforms showing P300 responses averaged across the three test runs. The downward arrows show P300, and the upward triangles show the following negative point; P300 amplitude was calculated between P300 and the negative point. Panels are ordered in terms of age at testing; the top row shows data for child (“C”) participants and the next two rows show data for adult (“A”) participants. The panels at bottom right show boxplots of P300 amplitude and latency across all three runs for NH (red) and CI listeners (blue); the boxes show the 25th and 75th percentiles, the error bars show the 10th and 90th percentiles, the filled circles show outliers, the horizontal lines show the median, and the white stars show the mean.

Figure 2 shows waveforms with the peak P300 response averaged across the three test runs for individual NH and CI listeners. Note that the intra-class correlation coefficient was 0.99 and 0.97 for P300 amplitude and latency, respectively, suggesting good test-retest reliability across the three runs. Because RM ANOVAs showed no significant effect of test run for NH or CI participants (*p* > 0.05 for all analyses), data were averaged across runs. Mean P300 amplitude was higher for the NH group (8.9 ± 3.5 μV) than for the CI group (3.2 ± 2.2 μV); mean P300 latency was shorter for the NH group (305 ± 23 ms) than for the CI group (338 ± 28 ms). *T*-tests showed that P300 amplitude was significantly higher for the NH than for the CI group [*t*(20) = 4.6, *p* < 0.001], and that P300 latency was significantly shorter for the NH than for the CI group [*t*(20) = −3.1, *p* = 0.006].

**Figure 3:**
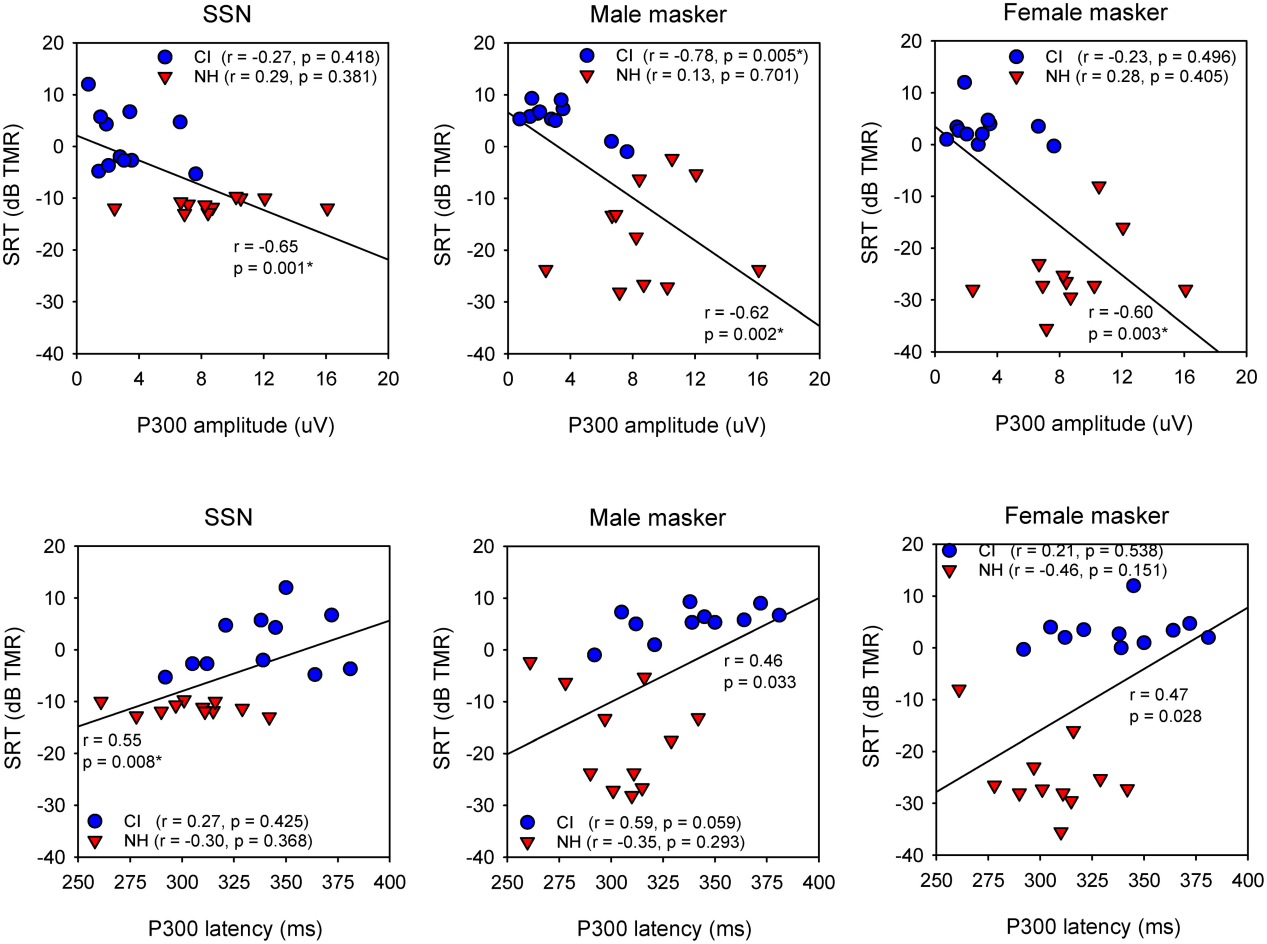
**(Top)** Scatter plots of SRTs with SSN (left) or with a competing male (middle) or female talker (right) as a function of P300 amplitude, for the NH (red triangles) and CI listeners (blue circles). The diagonal line shows the linear regression across all data; the correlation coefficient and *p* value are shown near the line. Correlation coefficients and *p* values are shown for the CI data and NH data in the legend. Significant relationships after Bonferroni correction for multiple comparisons are indicated by asterisks. **(Bottom)** Same as top, but for SRTs as a function of P300 latency.

Figure 3 shows SRTs with SSN or with a competing male or female talker for the NH and CI groups as a function of P300 amplitude and latency; each data point shows the mean across three test runs. When all NH and CI data were combined, and after Bonferroni correction for multiple comparisons (adjusted *p* = 0.016), Pearson correlation analysis showed significant relationships between P300 amplitude and SRTs with SSN (*r* = −0.65, *p* = 0.001), and with the male (*r* = −0.62, *p* = 0.002) and female maskers (*r* = −0.60, *p* = 0.003). A significant relationship was observed between P300 latency and SRTs with SSN (*r* = 0.60, *p* = 0.008), but not for SRTs with the male or female masker. For the CI group, Pearson correlation analysis showed a significant relationship only between P300 amplitude and SRTs with the male masker (*r* = −0.78, *p* = 0.005); the correlation remained significant after controlling for age at testing, duration of deafness, and CI experience (*r* = −0.81, *p* = 0.016). No significant correlations were observed between P300 amplitude and SRTs with SSN or with the female masker, or between P300 latency and SRTs with any of the maskers. For the NH group, no significant relationships were observed between P300 amplitude or latency and SRTs with any of the maskers. For the CI group, a significant correlation was observed between P300 amplitude and unaided PTA thresholds (across all frequencies; *r* = −0.87, *p* < 0.001); there were no significant correlations between P300 amplitude and aided PTA thresholds. Significant correlations were observed between P300 latency and unaided PTA thresholds (*r* = 0.67, *p* = 0.025) and aided PTA thresholds (*r* = 0.68, *p* = 0.021). Note that statistical power was >0.80 for all of the above correlations, except for P300 latency vs. unaided PTA thresholds (power = 0.63) or aided PTA thresholds (power = 0.65).

## Discussion

Consistent with previous studies (e.g., Kubo et al., 2001; Beynon et al., 2005; Obuchi et al., 2012; Soshi et al., 2014; Grasel et al., 2018; Han et al., 2020), P300 amplitudes were significantly larger and latencies were significantly shorter for the NH group than for the CI group. For the present bimodal CI listeners, mean P300 amplitude and/or latency values were comparable to those observed in previous studies with CI listeners (e.g., Iwaki et al., 2004; Sasaki et al., 2009; Grasel et al., 2018; Abrahamse et al., 2021; Calderaro et al., 2020; Van Yper et al., 2020). P300 responses were elicited in all CI participants, consistent with Obuchi et al. (2012).

Mean SRTs for all maskers were lower (better) for the NH group than for the CI group, and values were comparable to those in previous studies using similar methods and stimuli (Tao et al., 2018; Zhang et al., 2020). Different from previous CI studies that showed lower SRTs in SSN than in competing speech (e.g., Cullington and Zeng, 2008; Croghan and Smith, 2018; Tao et al., 2018; Liu et al., 2019), there was no significant difference in SRTs between the SSN and competing speech maskers within the CI group. Note that CI listeners were tested while wearing contralateral hearing aids, which likely aided in segregation of competing speech, thereby reducing the deficit relative to SSN.

Across all NH and CI listeners, significant correlations were observed between P300 amplitude and SRTs with the SSN, male, and female maskers; a significant correlation was also observed between P300 latency and SRTs with SSN. These correlations were largely driven by across-group differences in speech performance and P300 responses. In general, higher P300 amplitude and shorter P300 latency were associated with better masked speech recognition.

In the NH group, there were no significant correlations between P300 responses and SRTs with any of the maskers. In the CI group, a significant correlation was observed only between P300 amplitude and SRTs with the male masker, the most challenging listening condition with the greatest informational masking. The correlation between P300 amplitude and SRTs with the male masker suggests some common relation to informational masking, a central auditory process. With the female masker, informational masking was reduced, and SSN produced largely energetic masking. Given the correlations between unaided PTA thresholds and P300 amplitude and latency and between aided PTA thresholds and P300 latency, differences in P300 response across CI listeners may have represented differences in segregation of the competing male talkers with residual acoustic hearing that provided low-frequency pitch cues.

Different from Soshi et al. (2014), we observed a significant correlation between P300 amplitude and SRTs with the male masker, but not between P300 amplitude and SRTs with SSN. Differences in cortical measure stimuli (1 vs. 2 kHz contrasts; consonant contrast), speech tests, methods, and CI patients (bimodal vs. CI-only listening) may have contributed to differences in results across studies. The 1 and 2 kHz stimuli used for ERP recording were presented at 20–30 dB above the aided thresholds, meaning that the aided acoustic hearing should have contributed to the response.

In the present study, ERPs and speech performance were measured only with bimodal listening. Some studies have shown greater P300 response and speech performance with bimodal than with CI-only listening (e.g., Iwaki et al., 2004; Sasaki et al., 2009). Interestingly, Wedekind et al. (2021) found no significant difference in P300 response between the NH ear and the CI ear in unilaterally deaf CI recipients; speech recognition in noise was better with the CI on than off. While it was not directly measured in Wedekind et al. (2021), speech performance would be expected to be much poorer with the CI ear alone than with the NH ear alone (e.g., Galvin et al., 2019). It is unclear why the P300 response would be similar across ears when speech performance would be different. As shown in Figure 3, significant relationships were observed between P300 amplitude and masked SRTs, presumably due to the underlying spectro-temporal resolution that was much better for NH than for CI listeners. However, some caution is warranted regarding the correlational analyses, given the limited number of participants and test runs. ERPs and speech performance were not measured with the acoustic-hearing ear alone or the CI ear alone in this study. It is possible that strong P300 responses may have been elicited within the acoustic-hearing ear alone, despite the expectedly poor speech performance. In future studies, it would be worthwhile to collect ERPs and speech performance with each ear alone and both ears together to better understand how the peripheral representations might affect the relationship between ERPs and speech performance.

The present results show some evidence that ERPs may be a useful objective measure to predict complex perception such as segregation of competing speech. However, eliciting P300 also requires a behavioral component in the oddball presentation, and the magnitude of the response may depend on the strength of the stimulus contrast. Obuchi et al. (2012) showed increasing P300 amplitude in CI listeners as the stimulus frequency contrast was increased from 1.5 to 4 kHz. Depending on the acoustic-to-electric frequency allocation and the electrode-neural interface (electrode position relative to healthy neurons), small contrasts (e.g., 1 vs. 1.5 kHz) may be perceived differently among CI listeners. The 1 vs. 2 kHz contrast in this study appeared to be sufficiently large to be discriminated by the present MED-EL CI users, most likely resulting in stimulation of electrodes 6 and 8, given the default frequency allocation. Note that there may have been some contribution from residual acoustic hearing for discrimination of the stimuli contrast.

## Conclusions

Auditory ERPs and speech recognition in steady noise or competing speech were measured in NH and bimodal CI listeners. P300 amplitude was larger and latency was shorter in the NH group than in the CI group. Similarly, speech performance was better for the NH group than for the CI group. Significant correlations were observed across all participants between P300 amplitude and SRTs with steady noise and the male and female maskers. Within the CI group, P300 amplitude was significantly correlated with SRTs with the male masker, suggesting some relation between cortical response and informational masking.

## Data Availability Statement

The raw data from the study are included in the supplementary material; further inquiries can be directed to the corresponding author.

## Ethics Statement

The studies involving human participants were reviewed and approved by the Ethical Committee from the First Affiliated Hospital of Soochow University (Approval number 2021122). All participants provided written informed consent before participating in the study; approval was obtained for pediatric CI and NH participants from their parents or adult next of kin.

## Author Contributions

D-DT: study design, data analysis, and writing of manuscript. Y-MZ and DZ: data analysis and writing of manuscript. JG: data analysis, data visualization, and writing of manuscript. HL, WZ, and MX: study supervision. J-SL: study design. All authors contributed to the article and approved the submitted version.

## Funding

This work was supported by the National Natural Science Foundation of China (81970877), the Jiangsu Provincial Natural Science Foundation—Outstanding Youth Foundation (BK20200054), the Jiangsu Provincial Key Research and Development Program Special Funds (BE2019670), and the Suzhou Science and Technology Project (SYS2019049).

## Conflict of Interest

The authors declare that the research was conducted in the absence of any commercial or financial relationships that could be construed as a potential conflict of interest.

## Publisher's Note

All claims expressed in this article are solely those of the authors and do not necessarily represent those of their affiliated organizations, or those of the publisher, the editors and the reviewers. Any product that may be evaluated in this article, or claim that may be made by its manufacturer, is not guaranteed or endorsed by the publisher.
